# 1447. Cluster of Carbapenem-resistant Acinetobacter baumannii in the Context of Local Community Transmission

**DOI:** 10.1093/ofid/ofad500.1284

**Published:** 2023-11-27

**Authors:** Malik Darwish, Angel Desai, Marinell Catalan, Timothy Wilson, Colin McGlynn, Jennifer Suhd-Brondstatter, Allison Dow, Amy Kingsley, Mary Reilly, Stuart H Cohen

**Affiliations:** University of California Davis Medical Center; University of California Davis Medical Center; University of California Davis Medical Center; University of California Davis Medical Center; University of California Davis Medical Center; University of California Davis Medical Center; University of California Davis Medical Center; University of California Davis Medical Center; University of California Davis Medical Center; University of California, Davis, Sacramento, CA

## Abstract

**Background:**

Carbapenem-resistant *Acinetobacter baumannii* is increasingly being recognized as a nosocomial pathogen of concern. We report on a cluster of infections at a tertiary-care hospital in the context of local community outbreaks.

**Methods:**

Between July and October 2021, we identified an increase in carbapenem-resistant *Acinetobacter baumannii* infections at our medical center. We conducted epidemiological investigation followed by whole-genome sequencing of clinical isolates in tandem with the Public Health Department, who was monitoring ongoing regional long-term care facility outbreaks.

**Results:**

Of 9 patients with carbapenem-resistant *Acinetobacter baumannii* isolates, 8 were sent for whole genome sequencing (WGS) which revealed two clonally related groups; Clone 1 accounted for four cases, Clone 2 accounted for two, and the remaining two were unrelated to the other isolates. Half of the Clone 1 cases and all of the Clone 2 cases presented from the same two long-term care facilities, likely reflecting a known regional outbreak. The other two Clone 1 cases presented from the community and were suspected to be healthcare-acquired prior to WGS confirmation, although no definite route of transmission was ultimately identified. In response, we performed audits on the affected wards to improve targeted infection prevention practices, including enhanced cleaning and disinfection of respiratory therapy equipment. Concurrently, we initiated a surveillance system to screen all patients upon admission from long-term care facilities for colonization via rectal swabs cultured on *Acinetobacter*-selective media. Subsequent point prevalence testing revealed no additional healthcare-associated infections.

**Conclusion:**

We identified a cluster of carbapenem-resistant *Acinetobacter baumannii* cases that predominantly reflected ongoing outbreaks at long-term care facilities in the community. Two likely healthcare-associated cases were also identified through whole genome sequencing, prompting infection prevention measures and targeted screening. Our experience highlights the necessity of a robust surveillance system and mitigation efforts to prevent transmission of healthcare-associated infections.

**Disclosures:**

**All Authors**: No reported disclosures

